# Supplementary material for: The cellular and KSHV A-to-I RNA editome in primary effusion lymphoma and its role in the viral lifecycle
Source: Nat Commun. 2023 Mar 13;14:1367. doi: 10.1038/s41467-023-37105-8 (PMC10011561; doi:10.1038/s41467-023-37105-8)

Supplementary Information

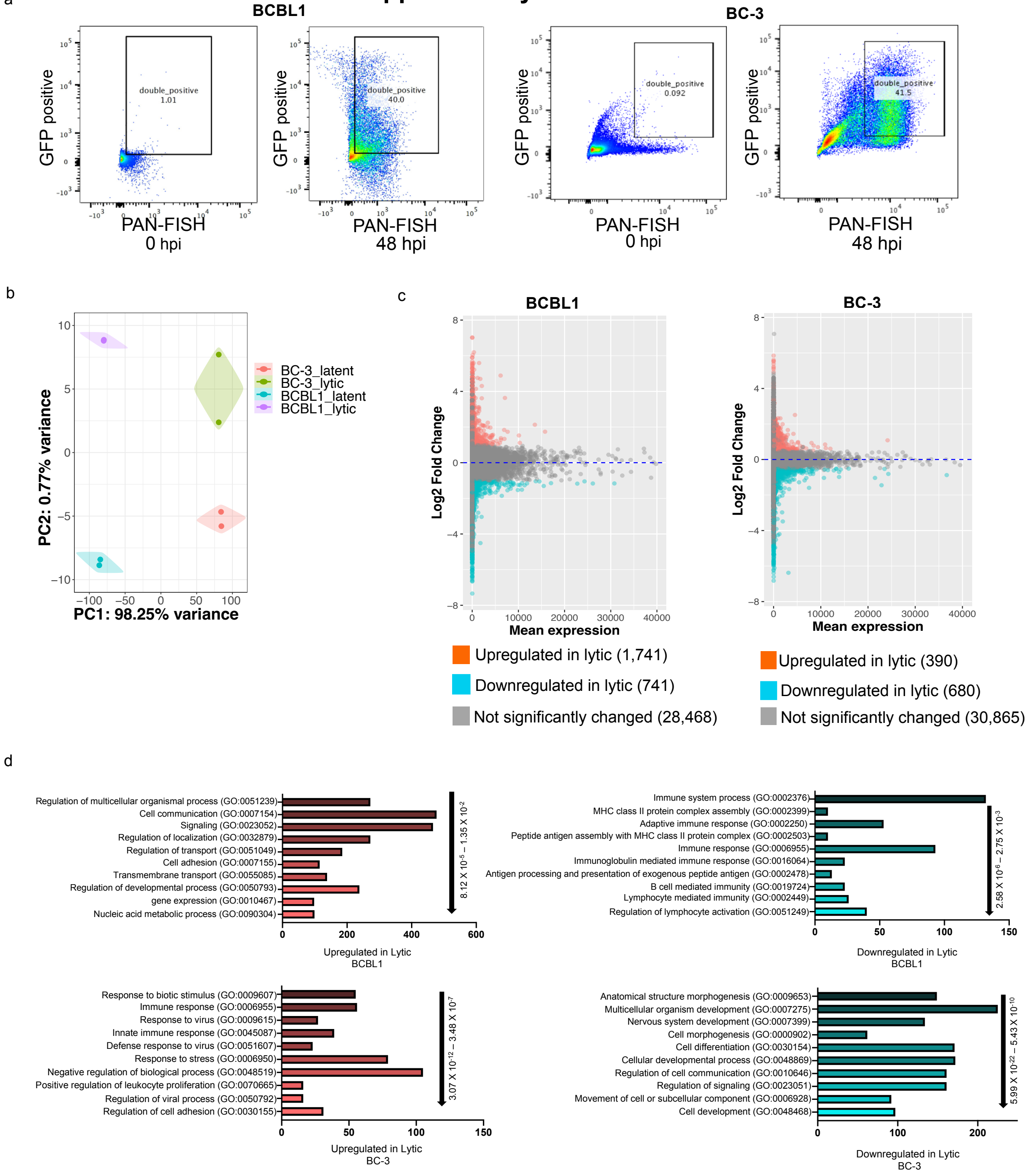

Supplementary Figure 1: Characterization of reporter PEL cells to study KSHV latent and lytic transcriptomes

a, Flow cytometry assessing latent and lytic BCBL1 (left) and BC-3 (right) cells by GFP and PAN FISH-FLOW. **b**, Principal component analysis of the RNA-seq from latent and lytic infected BCBL1 and BC-3 cells. **c,** Differential gene expression analysis of latent and lytic infected BCBL1 (left) and BC-3 (right) cells. Red and blue dots represent up- (P-adj < 0.05, log2 fold > 1) and downregulated (P-adj < 0.05, log2 fold < -1) genes in lytic infected cells compared to latent infected cells. The P-adj values were calculated using the Wald test (default testing in Deseq2) and no further adjustments were made. The number of genes that are up- and downregulated are indicated within parenthesis. Grey dots represent genes that are not significant (P-adj > 0.05) between two biological replicates of latent and lytic RNA seq. **d**, Gene ontology analysis of up- (left) and downregulated (right) genes in lytic reactivation in BCBL1 and BC-3 Cells.

a

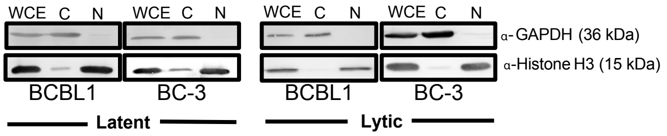

b

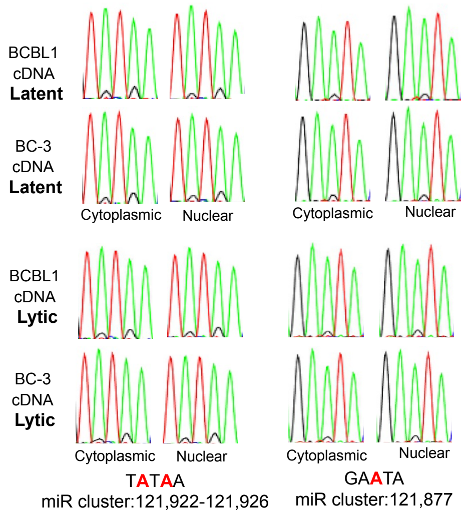

c

Chromosomal position	BCBL1				BC-3			
	**Latent**		**Lytic**		**Latent**		**Lytic**	
	Cytoplasm	Nuclear	Cytoplasm	Nuclear	Cytoplasm	Nuclear	Cytoplasm	Nuclear
miR Cluster:121,923	8.50%	7.80%	7.80%	7.80%	8.30%	8.10%	5.50%	8.10%
miR Cluster:121,925	16%	15%	14.20%	11.70%	15%	12%	13.50%	12.80%
miR Cluster:121,877	9.60%	9.10%	9.10%	9.10%	9.10%	9.20%	6.30%	8.80%

Supplementary Figure 2: Editing of miRNA-12-4 stem and seed region in nuclear and cytoplasmic fractions in PEL cells

a, Western blot analysis of indicated proteins from subcellular fractionation. WCE, whole cell extract; C, cytoplasmic; N, nuclear. N=2 biologically independent experiments. **b**, Sanger sequencing chromatograms of cDNA amplified from RNA extracted from subcellular fractionation. The chromosomal coordinates (GQ994935.1) for each editing site are listed below the chromatogram. The nucleotides at each position are represented with a different color (Green = Adenosine, Black = Guanosine, Blue = Cytidine, Red = Thymidine). Latent cells refer to the untreated PEL cells and lytic cells refer to the isolated GFP positive cells at 48 hpi. Source data are provided as a Source Data file.

a

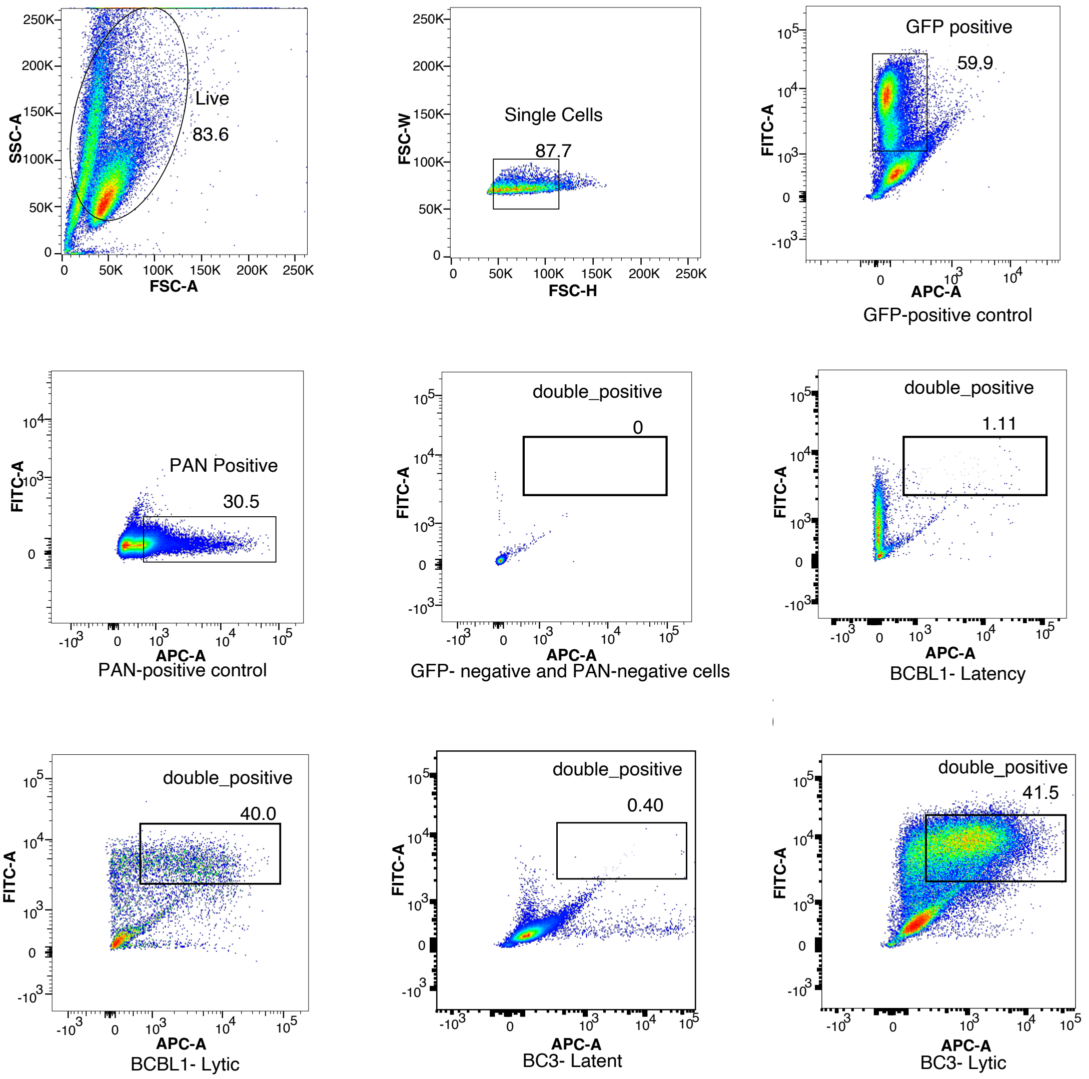

b

Number of viral reads	Latent rep 1	Latent rep 2	Lytic rep 1	Lytic rep 1
BCBL1	21,872	22,291	682,337	638,258
BC3	41,502	27,545	249,236	595,397

c

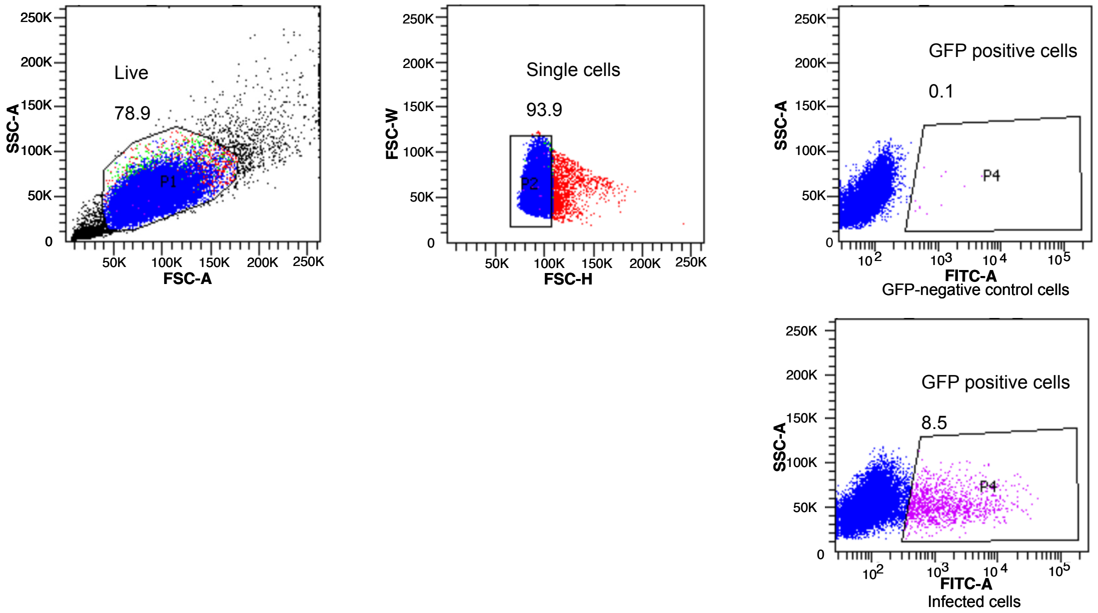

Supplementary Figure 3: Strategy to isolate lytic reactivated PEL cells used in our study

a, Gating strategy used in flow cytometry to isolate lytic BCBL1 and BC3 cells (related to Fig. 1a). SSC (side scatter) and FSC (forward scatter) were used to select the single cell population and dead cells from this population were excluded. Two populations of TREx-BCBL1-PAN-GFP cells were reactivated with Dox for 48 hours. One of them were hybridized with PAN Alexa-Fluor 647 probe and used as PAN positive control. The other population that was not hybridized with PAN probe was used as GFP positive control. Latent TREx-BCBL1 cells were used as GFP negative and PAN negative cells. FITC refers GFP and APC refers PAN Alexa-Fluor 647. b, The number of RNA-seq reads aligned to KSHV genome (GQ994935.1) in each of our samples. c, Gating strategy used in flow cytometry to quantify the infected HEK293T and HUVECs in Fig. 5d. SSC (side scatter) and FSC (forward scatter) were used to select the single cell population and dead cells from this population were excluded. Uninfected HEK293T cells were used as GFP negative control. FITC refers GFP signal.

Supplementary Table S1

Chromosomal position	Gene Name	Strand	% of editing in Latent	% of editing in Lytic	Genomic location	Predicted amino acid change	Function
**BCBL1**							
92638	K11	T>C		13.5	CDS	S-to-G	vIRF homolog
117809	K12	T>C		28.6	CDS	S-to-G	Induce tumorigenic growth
119815	Upstream of K12	T>C	18.2				Induce tumorigenic growth
120882	miRNA cluster	T>C	13.5	10.8			
121923	miRNA cluster	T>C	22.8	11.9			
121925	miRNA cluster	T>C	8.5				
121877	miRNA cluster	T>C	5.5	3.5			
125978	ORF 73	T>C		25			Latency maintenance
115755	Upstream of ORF67/67A	T>C		15.4			Encodes a tegument protein, homologous to the Epstein-Barr virus gene BFRF1
125953	ORF73	T>C	57.1	57.1	CDS	Q-to-Q	Latency maintenance
126203	ORF73	T>C	50	71.4	CDS	Q-to-R	Latency maintenance
126233	ORF73	T>C		60	CDS	Q-to-R	Latency maintenance
126268	ORF73	T>C		93.3	CDS	Q-to-Q	Latency maintenance
73502	ORF50	A>G	14	35	CDS	E-to-G	Lytic reactivation
121858	Repeats	A>G		28.6			
122427	Repeats	A>G		99.1			
121924	Repeats	A>G		22.2			
**BC-3**							
92638	K11	T>C		9.2391304	CDS	S-to-G	vIRF homolog
117580	Upstream of K12	T>C		2.4144869			Induce tumorigenic growth
117809	K12	T>C	13.3333333	28.8172043	CDS	S-to-G	Induce tumorigenic growth
120123	Repeats	T>C	75	94.1176471			Induce tumorigenic growth
120882	miRNA cluster	T>C	9.7826087	18.5393258			
121034	miRNA cluster	T>C		4.1884817			
121852	miRNA cluster	T>C		5.0980392			
121877	miRNA cluster	T>C	5	6.8669528			
121923	miRNA cluster	T>C	6.3218391	14.1463415			
121925	miRNA cluster	T>C	4	9.8039216			
121729	miRNA cluster	T>C		3.2110092			
29208	Downstream of ORF17	T>C		13.3333333			Capsid scaffolding protein
29243	Downstream of ORF17	T>C		15.3846154			Capsid scaffolding protein
29254	Downstream of ORF17	T>C		15			Capsid scaffolding protein
29324	Downstream of ORF17	T>C		12.5			Capsid scaffolding protein
73502	ORF50	A>G	60	72	CDS	E-to-G	Lytic reactivation
73483	ORF50	A>G	80.6451613	48.8095238	CDS	A-to-A	Lytic reactivation
74048	ORF50	A>G	55.2631579	52.8571429	CDS	H-to-R	Lytic reactivation
125938	ORF73	T>C		94.4444444	CDS	Q-to-Q	Latency maintenance
125953	ORF73	T>C	40	46.1538462	CDS	Q-to-Q	Latency maintenance
125978	ORF73	T>C		27.2727273	CDS	Q-to-R	Latency maintenance
125993	ORF73	T>C		18.1818182	CDS	Q-to-Q	Latency maintenance
126083	ORF73	T>C	96.6666667	95.1219512	CDS	Q-to-Q	Latency maintenance
126098	ORF73	T>C		98.8095238	CDS	Q-to-Q	Latency maintenance
126268	ORF73	T>C	8.8235294	10.8108108	CDS	Q-to-Q	Latency maintenance
125923	ORF73	T>C		96.5811966	CDS	Q-to-Q	Latency maintenance
126023	ORF73	T>C		7.9646018	CDS	Q-to-R	Latency maintenance
126253	ORF73	T>C	94.1176471	97.3333333	CDS	Q-to-Q	Latency maintenance
121858	Repeats	A>G		10	Repeats		
121924	Repeats	A>G		8	Repeats		

Supplementary Table S1: Latent and lytic KSHV editomes in PEL (related to Fig. 3a).

Edited sites within the KSHV transcriptome of latent and lytic infected BCBL1 and BC-3 cells . Sites are listed by chromosome and nucleotide position, gene name and the identified change in columns A, B and C respectively. Average percentage of editing in latent and lytic infected cells are listed in columns D and E respectively. The genomic location, predicted amino acid change (if the site maps to CDS) and reported function are listed in columns F, G and H respectively.

CDS- coding sequence

Supplementary Table S2

miRNA Taqman Probes		
**miRNA**	**Assay number**	**Cat#**
KSHV-miRNA-K12-4-3p	197240_mat	4427975
KSHV-miRNA-K12-4-5p	197234_mat	4427975
KSHV-miRNA-K12-9*	462207_mat	4440885
RNU48	001006	4427975
**qPCR primers**	**Sequnce 5'-3'**	
adar1_F	GACGCTTGTCTCCTTAGTCTTCCCGGATTG	
adar1_R	CATCTGACCCGTGCTATTTGCTGTCGTGTG	
adar2_F	GTGTAAGCACGCGTTGTACTGTCGCTGG	
adar2_R	GGTACTCCTTTGCCGCCAGCTTGGAC	
18S_rRNA_F	GTAACCCGTTGAACCCCATT	
18S_rRNA_R	CCATCCAATCGGTAGTAGCG	
KSHV_ORF59_F	ACAGTCACCGTTTGGTCCTC	
KSHV_ORF59_R	TGTACTCGACGCTGGCATAG	
KSHV_ORF57_F	TGGACATTATGAAGGGCATCCTA	
KSHV_ORF57_R	CGGGTTCGGACAATTGCT	
KSHV_ORF50_F	GAGTCCGGCACACTGTACC	
KSHV_ORF50_R	AAACTGCCTGGGAAGTTAACG	
KSHV_ORF45_F	GGGATGGGTTAGTCAGGATG	
KSHV_ORF45_R	CCTCGTCGTCTGAAGGTGA	
**siRNA**		
ADAR1 pool	Dharmacon Human ADAR1 pool	L-008630-00-0005
control pool	Dharmacon Human Non target pool	L-001810-10-05
**Editing assay**	**PCR primers**	
ORF50	gcgtttattagtacgtggcaggccg	
	gtaacgcattgcggtggttgaaattgc	
miRNA cluster	gaccggcaagttccaggcatcctaag	
	gcctgtaatgggctatcacattctgaggac	
Kaposin A	cagtgcgcgcccgttgcaac	
	ggatagaggcttaacggtgtttgtggc	
NOP14	cagcctggctacacaggaaggcg	
	ctacacatgaaggcaaacgtccgtg	
AJUBA	cgtaagccactgtgtctggcctagtgtatg	
	cattctcagacagataaggagctctcctag	
MAVS	gcctgtgaaccacagcttatcacatgtctg	
	ctattatagcaggtggcagatgttggcaca	
**Northern Blot**		
7SK	AGAAAGGCAGACTGCCACA	
miRNA-K12-4-3p	TCAGCTAGGCCTCAGTATTCTA	
**FISH FLOW**		
PAN	ACAAATGCCACCTCACTTTGTCGC	

Supplementary Table S2: Oligonucleotides used in study.

Supplementary Table S3

Editing levels related to Fig 1h			
Gene Name	Chromosomal position	Editing in BCBL1	Editing in BC3
NOP14	Chr 4: 2,938,437	15.60%	11%
	Chr 4: 2,938,420	11.50%	12.50%
	Chr 4: 2,938,358	20.00%	12.80%
AJUBA	Chr 14: 22,972,941	62.50%	50%
	Chr 14: 22,972,942	71.80%	60%
	Chr 14: 22,972,943	39.30%	30%
	Chr 14: 22,972,932	25.00%	22.80%
	Chr 14: 22,972,905	33.00%	29.10%
	Chr 14: 22,972,911	26.90%	20.00%
	Chr 14: 22,972,912	89.20%	88.40%
	Chr 14: 22,972,913	93.70%	92.00%
MAVS	Chr 20: 3,872,359	18.50%	0%
	Chr 20: 3,872,364	17.80%	0%
	Chr 20: 3,872,338	12.80%	0%
	Chr 20: 3,872,340	7.50%	0%

Editing levels related to Fig 3c		
Gene Name	Chromosomal position	Editing in BCBL1
Kaposin	117,809	51.50%
miR-K12-4	121,923	18.20%
miR-K12-4	121,925	23.10%
miR-K12-4	121,877	21.80%
ORF50	73,502	24.20%

Editing levels related to Fig 4b						
Gene Name	Chromosomal position	Editing in BC-3	Editing in JSC-1	Editing in BC-5	Editing in BC-1	Editing in iSLK-bac16
miR-K12-4	121,923	12.50%	5.10%	7.80%	5.10%	5.10%
miR-K12-4	121,925	20%	12.50%	16.60%	15.60%	16.00%
miR-K12-4	121,877	11.70%	5.40%	13.50%	8.50%	7.90%

Editing levels related to Fig 4c			
Gene Name	Chromosomal position	Editing in control KD	Editing in ADAR1 KD
miR-K12-4	121,923	20.00%	0%
miR-K12-4	121,925	25%	0%
miR-K12-4	121,877	23.10%	0%

Supplementary Table S3: Quantification of editing sites in Sanger sequencing chromatograms (related to Fig. 1h, 3c, 4b and 4c).

The heights of A and G peaks at the edited sites in Sanger sequencing chromatograms and the percentage of editing was quantified using the equation (peak height of G/(G+A)*100).

Uncropped Images for Supplementary Fig. 2a

BCBL1

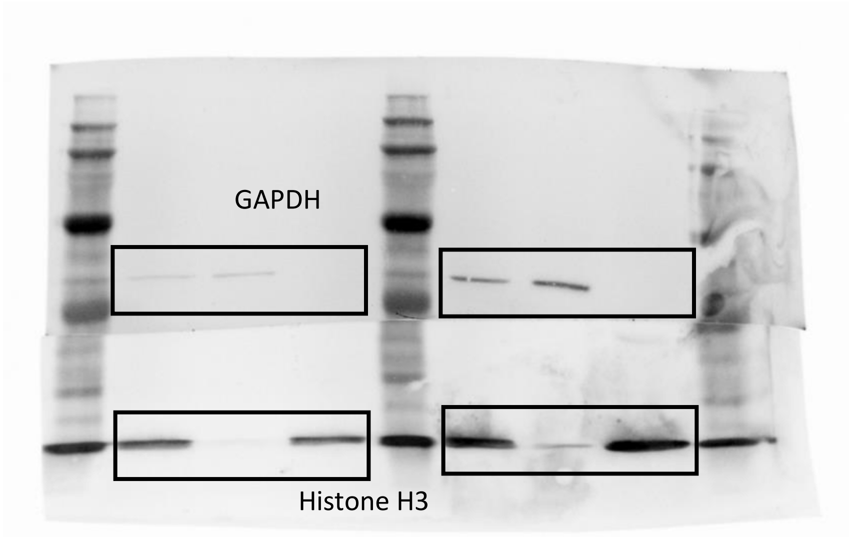

BC-3

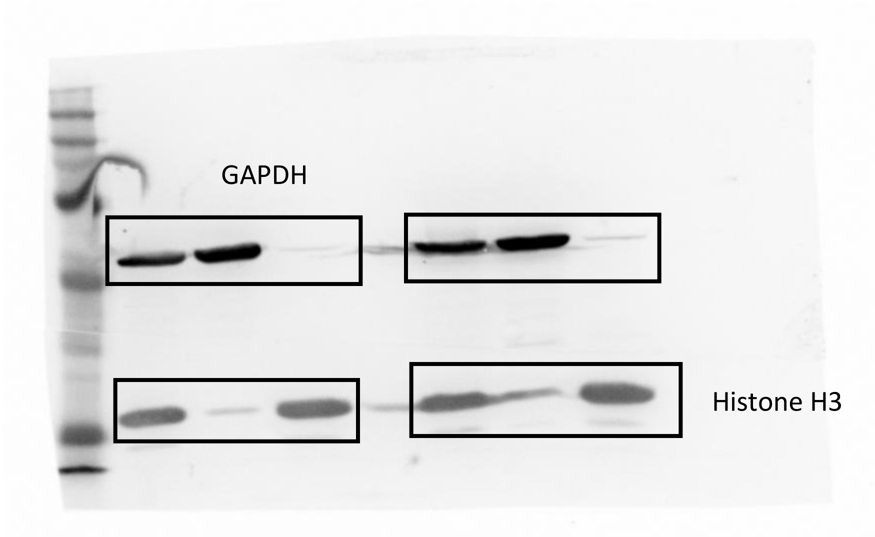

Supplement: Supplementary file 1 — Supplementary Information [file 41467_2023_37105_MOESM1_ESM.pdf]
