# Supplementary material for: The cellular and KSHV A-to-I RNA editome in primary effusion lymphoma and its role in the viral lifecycle
Source: Nat Commun. 2023 Mar 13;14:1367. doi: 10.1038/s41467-023-37105-8 (PMC10011561; doi:10.1038/s41467-023-37105-8)
Supplement: Supplementary file 4 — Description of Additional Supplementary Files [file 41467_2023_37105_MOESM4_ESM.pdf]

**Title: Supplementary data 1:**

**Description: Latent and lytic host editomes in PEL (related to Fig. 1d).** RNA-seq reads from isolated latent and lytic infected BCBL1 and BC-3 cells were analyzed by SAILOR, a previously published software program that predicts A-to-I editing sites. High confidence editing sites (>0.99 confidence, > 5 reads per site) identified are listed by chromosome and nucleotide position (column A). Fraction of editing for each site was calculated by the number of reads containing G divided by the total number of reads at a given site. The number of reads covering an individual nucleotide (coverage), type of nucleotide change (A-to-G or T-to-C) and fraction of editing for each identified site are listed in column B, C and D, respectively. Calculated confidence and strand specificity are listed in columns E and F, respectively. Identified high confidence sites were assigned to annotated genes using gencode.v39 annotations. gene ID, biotype, gene name and the genomic location are listed in columns G, H, I and J respectively.

**Title: Supplementary data 2:**

**Description: Quantification of editing in control and SOX overexpressing HEK-293T cells(related to Fig. 2n).**

Edited sites from control and SOX overexpressing HEK-293T cells were overlapped to identify common sites that are edited in both datasets. Sites are listed by chromosome and nucleotide position (column A). Percentage of editing in control replicate 1, replicate 2 and average of these two are listed in columns B, C and D respectively. Similarly, Percentage of editing in SOX overexpressing replicate 1, replicate 2 and average of these two are listed in columns E, F and

G respectively. The calculated difference in editing from latent to lytic infection is listed in column H.
