# Supplementary material for: The cellular and KSHV A-to-I RNA editome in primary effusion lymphoma and its role in the viral lifecycle
Source: Nat Commun. 2023 Mar 13;14:1367. doi: 10.1038/s41467-023-37105-8 (PMC10011561; doi:10.1038/s41467-023-37105-8)
Supplement: Supplementary file 5 — Reporting Summary [file 41467_2023_37105_MOESM5_ESM.pdf]

nature portfolio

Corresponding author(s): John Karijolich

Last updated by author(s): 2/24/2023

Reporting Summary

Nature Portfolio wishes to improve the reproducibility of the work that we publish. This form provides structure for consistency and transparency in reporting. For further information on Nature Portfolio policies, see our Editorial Policies and the Editorial Policy Checklist.

Statistics

For all statistical analyses, confirm that the following items are present in the figure legend, table legend, main text, or Methods section.

n/a	Confirmed	
☐	☒	The exact sample size (*n*) for each experimental group/condition, given as a discrete number and unit of measurement
☐	☒	A statement on whether measurements were taken from distinct samples or whether the same sample was measured repeatedly
☐	☒	The statistical test(s) used AND whether they are one- or two-sided<br>*Only common tests should be described solely by name; describe more complex techniques in the Methods section.*
☒	☐	A description of all covariates tested
☐	☒	A description of any assumptions or corrections, such as tests of normality and adjustment for multiple comparisons
☐	☒	A full description of the statistical parameters including central tendency (e.g. means) or other basic estimates (e.g. regression coefficient) AND variation (e.g. standard deviation) or associated estimates of uncertainty (e.g. confidence intervals)
☐	☒	For null hypothesis testing, the test statistic (e.g. F, t, r) with confidence intervals, effect sizes, degrees of freedom and P value noted<br>*Give P values as exact values whenever suitable.*
☒	☐	For Bayesian analysis, information on the choice of priors and Markov chain Monte Carlo settings
☒	☐	For hierarchical and complex designs, identification of the appropriate level for tests and full reporting of outcomes
☒	☐	Estimates of effect sizes (e.g. Cohen's *d*, Pearson's *r*), indicating how they were calculated

Our web collection on statistics for biologists contains articles on many of the points above.

Software and code

Policy information about availability of computer code

Data collection	A previously published software SAILOR was used to identify A-to-I edited sites.

Data analysis	Flow cytometry data was analyzed with FlowJo X (9.6.4).<br>High throughput RNA sequencing raw reads quality in fastq files were accessed by FastQC (v0.11.8, https://www.bioinformatics.babraham.ac.uk/projects/fastqc/). Raw reads were trimmed of adapters and aligned to the human genome (GRCh38.p13) and KSHV genome (GQ994935.1) using STAR (v2.7.3a). Uniquely aligned reads were used as inputs to run featureCounts (v.1.5.2) to map the reads to gencode.v39 annotations using [-s 2] flag. For differential gene expression, DESeq2 (v1.18.1) was run with raw read counts obtained from featureCounts. FeatureCounts was generated by Love, M. I., Huber, W. & Anders, S. Moderated estimation of fold change and dispersion for RNA-seq data with DESeq2. Genome Biol 15, 550, doi:10.1186/s13059-014-0550-8 (2014). Transcripts that have a significant difference in gene expression (P-adj < 0.05, using Benjamini–Hochberg correction) were differentially expressed. Gene Ontology analysis of the differentially expressed genes during lytic reactivation was obtained using http://geneontology.org/. To identify high confidence editing sites, uniquely aligned RNA reads were used as inputs for SAILOR (1.0.4). SAILOR was generated by Deffit, S. N. et al. The C. elegans neural editome reveals an ADAR target mRNA required for proper chemotaxis. Elife 6, doi:10.7554/eLife.28625 (2017). Sites with a confidence of ≥0.99 were chosen for downstream analysis. Annotation of high-confidence sites was performed with a custom Python script using gencode.v39 annotations. This custom python script is deposited in Github (https://github.com/yexiang2046/AIediting). In order to identify the neighbor nucleotides , the corresponding 5' and 3' neighbor nucleotides of the edited sites were retrieved using BEDTools (v2.30.0). Five-nucleotide stretches (centered on the edited adenosine) were randomly picked from human genome and were used as the background to determine the overrepresented and underrepresented nucleotides around the edited sites using Two Sample Logo (http://twosamplelogo.org).

For manuscripts utilizing custom algorithms or software that are central to the research but not yet described in published literature, software must be made available to editors and reviewers. We strongly encourage code deposition in a community repository (e.g. GitHub). See the Nature Portfolio guidelines for submitting code & software for further information.

Data

Policy information about availability of data

All manuscripts must include a data availability statement. This statement should provide the following information, where applicable:

- Accession codes, unique identifiers, or web links for publicly available datasets
- A description of any restrictions on data availability
- For clinical datasets or third party data, please ensure that the statement adheres to our policy

Sequencing data from this study have been deposited in SRA under project number PRJNA875094 (GEO under accession number GSE212350). Sequencing data (GEO under accession number GSE128866) from a previous study have been used to analyze the effect of SOX in RNA editing. High throughput sequencing reads were aligned to the human reference genome (gencode GRCh38.p13, https://www.ncbi.nlm.nih.gov/assembly/GCF_000001405.39/) and KSHV genome (GQ994935.1, https://www.ncbi.nlm.nih.gov/nuccore/GQ994935.1).
All the transcripts were summarized to biotypes annotated with GENCODE database (gencode.v39, ftp://ftp.ebi.ac.uk/pub/databases/gencode/Gencode_human/release_39/gencode.v39.chr_patch_hapl_scaff.annotation.gtf.gz). Gene ontology analysis on the differentially expressed genes were performed using http://geneontology.org/. Source data are provided with this paper. A reporting summary for this article is available as a Supplementary file.

Human research participants

Policy information about studies involving human research participants and Sex and Gender in Research.

Reporting on sex and gender	N/A
Population characteristics	N/A
Recruitment	N/A
Ethics oversight	N/A

Note that full information on the approval of the study protocol must also be provided in the manuscript.

Field-specific reporting

Please select the one below that is the best fit for your research. If you are not sure, read the appropriate sections before making your selection.

☒ Life sciences ☐ Behavioural & social sciences ☐ Ecological, evolutionary & environmental sciences

For a reference copy of the document with all sections, see nature.com/documents/nr-reporting-summary-flat.pdf

Life sciences study design

All studies must disclose on these points even when the disclosure is negative.

Sample size	To perform the statistics and calculate the standard deviation, at least three biologically independent experiments were performed. The number of biological replicates is indicated in the figure legends. Sample sizes were chosen to adjust for natural variation in experiment type

based on observed magnitude and variation within given samples.

Data exclusions	No data were excluded from the analysis
Replication	All relevant experiments were repeated at least three times. All replication attempts were successful.
Randomization	No randomization was performed, samples were treated according to the same protocols side-by-side with the respective controls
Blinding	Personnel handling the sequencing of RNA-seq data were blinded. Researchers were blinded to sample identity during imaging and data analysis.

Reporting for specific materials, systems and methods

We require information from authors about some types of materials, experimental systems and methods used in many studies. Here, indicate whether each material, system or method listed is relevant to your study. If you are not sure if a list item applies to your research, read the appropriate section before selecting a response.

Materials & experimental systems

n/a	Involved in the study
☐	☒ Antibodies
☐	☒ Eukaryotic cell lines
☒	☐ Palaeontology and archaeology
☒	☐ Animals and other organisms
☒	☐ Clinical data
☒	☐ Dual use research of concern

Methods

n/a	Involved in the study
☒	☐ ChIP-seq
☐	☒ Flow cytometry
☒	☐ MRI-based neuroimaging

Antibodies

Antibodies used	ADAR1 (E6X9R, Cell signaling technology, #81284s, Lot number 1, Rabbit monoclonal, 1:1000), Histone H3 (Millipore, #05-928, Lot number 2967216, clone A3S, Rabbit monoclonal, 1:1000), GAPDH (Proteintech, 60004-1-Ig, mouse monoclonal 1:5000), Alexa-Fluor 680-conjugated goat anti-mouse (#A28183, Lot 2491365 1:10,000), and Alexa-Fluor 680-conjugated goat anti-rabbit (#A27042, lot 2465353, 1:10,000)
Validation	Anti- ADAR1 antibody (Cell signaling technology) was validated in IP, WB, Confocal immunofluorescent analysis and Immunohistochemical analysis to detect ADAR1 by the manufacturer. Anti-Histone H3 Antibody, clone A3S (Rabbit Monoclonal Antibody) was validated in ChIP, WB to detect Histone H3 by the manufacturer (Millipore). Anti-GAPDH antibody (Proteintech) was validated by the manufacturer website for western blot, IP, immunofluorescence.

Eukaryotic cell lines

Policy information about cell lines and Sex and Gender in Research

Cell line source(s)	TREx-BCBL1-RTA and BC3-RTA cells were provided by Dr. Britt Galunsinger (UC Berkeley). TREx-BCBL1-RTA cells was generated by Nakamura, H. et al. Global changes in Kaposi's sarcoma-associated virus gene expression patterns following expression of a tetracycline-inducible Rta transactivator. J Virol 77, 4205-4220, doi:10.1128/jvi.77.7.4205-4220.2003 (2003). Wild type and miRNA-K12-4 deleted iSLK-bac16 cells were provided by Dr. RolF Renne (University of Florida) and generated by Jain, V. et al. A Toolbox for Herpesvirus miRNA Research: Construction of a Complete Set of KSHV miRNA Deletion Mutants. Viruses 8, doi:10.3390/v8020054 (2016). HEK293T and HUVECs were purchased from ATCC.
Authentication	Cell lines were obtained from investigators who generated them, and then passed through an intermediary, or generated in our lab. They are not further authenticated.
Mycoplasma contamination	All cell lines used in this study were mycoplasma free. Cell lines were routinely tested for mycoplasma by PCR.
Commonly misidentified lines (See ICLAC register)	No commonly misidentified cell lines were used in this study.

Flow Cytometry

Plots

Confirm that:

- [x] The axis labels state the marker and fluorochrome used (e.g. CD4-FITC).
- [x] The axis scales are clearly visible. Include numbers along axes only for bottom left plot of group (a 'group' is an analysis of identical markers).
- [x] All plots are contour plots with outliers or pseudocolor plots.
- [x] A numerical value for number of cells or percentage (with statistics) is provided.

Methodology

Sample preparation	Approximately 5X106 TREx-BCBL1-PAN-GFP and BC3-PAN-GFP cells were collected from latent and lytic (48 hpi with 2 µg/ml of Dox) infections and fixed in 4% (vol/vol) paraformaldehyde for 30 min at RT, washed with PBS-FISH buffer (1X PBS, 0.2 mg/ml RNase-free BSA) twice, and then permeabilized with 1X PBS containing 0.2% (vol/vol) Tween-20 for another 30 min at RT. The permeabilized cells were then hybridized with Alexa-Fluor 488 or Alexa-Fluor 647 labeled PAN anti-sense oligos (sequences in Supplementary Table 1) in HB 10% dx buffer (10% (wt/vol) dextran sulfate, 2× saline-sodium citrate (SSC), 10% (vol/vol) formamide, 1 mg/ml tRNA and 0.2 mg/ml BSA) at 37 °C overnight. After extensive washing with HBW buffer (2× SSC, 10% (vol/vol) formamide and 0.2 mg/ml RNase-free BSA) and cells were analyzed.
Instrument	cells were analyzed on BD Canto II instrument.
Software	Data were analyzed with FlowJo X software.
Cell population abundance	For cell sorting purity of the sorted population was determined by post-sort purity tests with the same settings. Abundance of the sorted portion was determined by the cut off gate, which was set to discriminate against non-lytic cells at ~80% of highest GFP positive cells.
Gating strategy	We first gated FSC-A AND SSC-A. After that we gated on single cells and GFP positive cells.

- [x] Tick this box to confirm that a figure exemplifying the gating strategy is provided in the Supplementary Information.
